# AMP-activated protein kinase is dispensable for maintaining ATP levels and for survival following inhibition of glycolysis, but promotes tumour engraftment of Ras-transformed fibroblasts

**DOI:** 10.18632/oncotarget.3738

**Published:** 2015-03-30

**Authors:** Joffrey Pelletier, Danièle Roux, Benoit Viollet, Nathalie M. Mazure, Jacques Pouysségur

**Affiliations:** ^1^ Institute for Research on Cancer and Ageing of Nice (IRCAN), University of Nice-Sophia Antipolis, CNRS UMR INSERM, Centre Antoine Lacassagne, Nice, France; ^2^ INSERM U1016, Institut Cochin, Paris, France; ^3^ CNRS UMR8104, Paris, France; ^4^ Université Paris Descartes, Sorbonne Paris Cité, Paris, France; ^5^ Centre Scientifique de Monaco (CSM), Monaco

**Keywords:** AMPK, AMPK-null cells, ATP, cancer cell, glycolysis

## Abstract

Lactic acid generated by highly glycolytic tumours is exported by the MonoCarboxylate Transporters, MCT1 and MCT4, to maintain pHi and energy homeostasis. We report that MCT1 inhibition combined with *Mct4* gene disruption severely reduced glycolysis and tumour growth without affecting ATP levels. Because of the key role of the 5′-AMP-activated protein kinase (AMPK) in energy homeostasis, we hypothesized that targeting glycolysis (MCT-blockade) in AMPK-null (*Ampk*^−/−^) cells should kill tumour cells from ‘ATP crisis’. We show that *Ampk*^−/−^-Ras-transformed mouse embryonic fibroblasts (MEFs) maintained ATP levels and viability when glycolysis was inhibited. In MCT-inhibited MEFs treated with OXPHOS inhibitors the ATP level and viability collapsed in both *Ampk*^+/+^ and *Ampk*^−/−^ cells. We therefore propose that the intracellular acidification resulting from lactic acid sequestration mimicks AMPK by blocking mTORC1, a major component of an ATP consuming pathway, thereby preventing ‘ATP crisis’. Finally we showed that genetic disruption of *Mct4* and/or *Ampk* dramatically reduced tumourigenicity in a xenograft mouse model suggesting a crucialrolefor these two actors in establishment of tumours in a nutrient-deprived environment. These findings demonstrated that blockade of lactate transport is an efficient anti-cancer strategy that highlights the potential in targeting *Mct4* in a context of impaired AMPK activity.

## INTRODUCTION

Dividing cancer cells rely extensively on glycolysis to maintain a high rate of proliferation [1-3]. Despite a lower yield of ATP than that of oxidative phosphorylation (OXPHOS), glycolysis generates precursors for the synthesis of nucleotides, amino acids, and membrane lipids [4]. Although exacerbated glycolysis is a common feature of any proliferative cell, genetic lesions driving cancer progression have a major impact on metabolism and promote glycolysis [5]. The dependency of cancer cells on glycolysis is amplified during tumour growth. Part of the tumour encounters an avascular microenvironment deficient in oxygen, i.e. hypoxic, as a consequence of rapid proliferation of cancer cells and aberrant neovasculature [6]. The transcription factor the Hypoxia-Inducible Factor 1 (HIF-1) [7], a key actor in cellular adaptation to hypoxia, induces a major shift in cellular metabolism toward glycolysis [6, 8, 9]. HIF-1, through the transcriptional upregulation of almost all the glycolytic enzymes together with inhibition of mitochondrial pyruvate dehydrogenase [10], creates an addiction to glycolysis of hypoxic tumour cells, which produce biomass as long as the major nutrients such as glucose are not depleted. So inhibition of glycolysis appears to be a promising avenue for the development of anti-tumour targeted therapies [11-15].

As a consequence of exacerbated glycolysis large amounts of lactic acid are produced. Our laboratory has been evaluating the anti-cancer approach of inhibiting lactic acid export. This approach leaded to intracellular accumulation of lactic acid associated with a severe drop in the intracellular pH (pHi), which results in inhibition of glycolysis [16, 17]. Lactate transport is carried out by four members of the monocarcarboxylate transporter (MCT) family (MCT1-4) [18], which have received revived attention in the context of cancer [19, 20]. They are H^+^/lactate bidirectional symporters with different affinities and tissue distribution. MCT1 is ubiquitous, whereas *MCT4*, a HIF-1-target gene [21], is strongly expressed in glycolytic tissues [22-24]. We and others showed that inhibition of MCTs or genetic disruption of MCT/basigin complexes leaded to a blockade in lactate transport and had anti-proliferative effects by reducing the glycolytic flux [15, 17, 25, 26]. However, although this approach severely restricted tumour growth it did not affect cancer cell viability unless the mitochondrial complex I was inhibited by biguanides [17, 25-27]. The major cause of resistance to cell death by inhibition of energy-producing pathways resides in the metabolic plasticity governed by the central guardian of the cellular ATP level, the AMP-activated protein kinase (AMPK) [2, 16, 27, 28]. AMPK is a highly conserved serine/threonine protein kinase involved in the control of energy homeostasis [28]. AMPK exists as a heterotrimeric protein comprising a catalytic α subunit and regulatory β/γ subunits. As a direct sensor of the AMP:ATP ratio, AMPK is rapidly activated by stresses that cause a reduction in ATP levels, such as glucose starvation [29], metabolic inhibitors [30], or muscle contraction [31, 32]. When activated, AMPK maintains a viable level of ATP, accelerating energy production, through increased glucose uptake [33], glycolysis [34], mitochondrial biogenesis [35], and mitophagy [36], while switching off energy consuming processes such as synthesis of proteins [37-40], glycogen [41] and fatty acids [42, 43]. However, although extensively studied, the regulation and function of AMPK remain only partially understood, especially in cancer.

We therefore hypothesized that genetic disruption of AMPK in a context of strong inhibition of glycolysis (MCT blockade) should sensitize tumour cells to death as a result of a deficit in maintenance of the ATP level. As no specific inhibitor for AMPK exists, a model of Ras-transformed murine embryonic fibroblasts (MEFs) genetically knocked out for the two catalytic subunits of AMPK (*Ampk^−/−^*), *Ampk*α*1* and *Ampk2* [44], was used. We first demonstrated that pharmacological inhibition of MCT1 combined with genetic knockout of *Mct4* (*Mct4^−/−^*) in Ras-transformed MEFs, lead to inhibition of glycolysis and proliferation *in vitro*. However, *Ampk^−/−^* MEFs preserved viable levels of ATP following acute inhibition of glycolysis. Moreover, AMPK was not capable of providing a survival advantage following severe inhibition of ATP production by glycolysis and OXPHOS. This unexpected finding suggested that AMPK is dispensable in regulating the plasticity of bioenergetic pathways. Finally we showed, using a xenograft tumour model, that the knockout of *Ampk* or *Mct4* (*Ampk^−/−^* or *Mct4^−/−^*) delayed tumour development while *Ampk^−/−^ Mct4^−/−^* MEFs severely impacted on tumour establishment. These *in vivo* studies suggest that combined inhibition of AMPK and MCT4 could be exploited as an anti-cancer strategy.

## RESULTS

### In the absence of an energy stress, genetic disruption of AMPK in MEFs does not affect glycolysis, OXPHOS or cell proliferation

**Figure 1:**
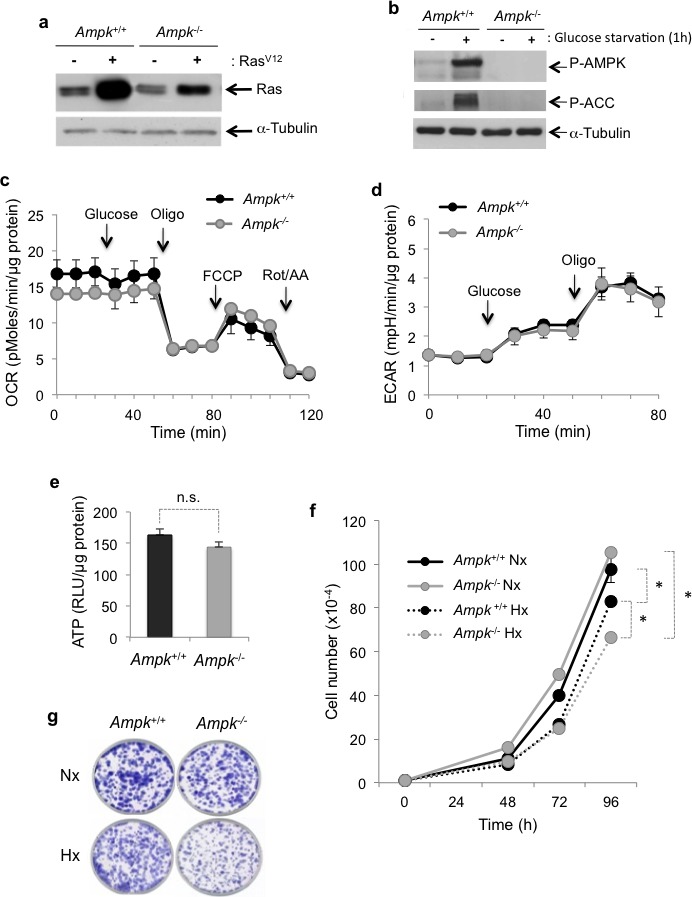
Metabolic characterization *in vitro* of Ras^v12^-transformed mouse embryonic fibroblasts (MEFs) expressing (*Ampk*^+/+^) or not (*Ampk^−/−^) Ampk* (**a**) Cell lysates of *Ampk*^+/+^ and *Ampk*^−/−^ MEFs not transformed (−) or transformed (+) by Ras^V12^ were analyzed by immunoblotting for Ras. (**b**) Cell lysates of *Ampk*^+/+^ and *Ampk*^−/−^ MEFs transformed by Ras^V12^ were analyzed by immunoblotting for phosphorylation of AMPK (Thr172) and ACC Ser79 after 1h of glucose starvation. (**c**) Oxygen consumption rate (OCR) was measured in real time with a Seahorse XF for *Ampk*^+/+^ (*Ampk*^+/+^) and *Ampk*^−/−^ (*Ampk*^−/−^) MEFs transformed by Ras^V12^ in normoxia. Glucose (10 mM), oligomycin (Oligo 1 μM), carbonilcyanide p-triflouromethoxyphenylhydrazone (FCCP 1μM) and rotenone/antimycine A (Rot/AA 1 μM) were injected at the indicated times. Mean±S.E.M. is representative of three independent experiments carried out in quadruplicate. (**d**) Extracellular acidification rate (ECAR) of normoxic *Ampk*^+/+^ (*Ampk*^+/+^) and *Ampk*^−/−^ (*Ampk*^−/−^) MEFs transformed by Ras^V12^ were evaluated with a Seahorse XF. Glucose (10 mM) and oligomycin (Oligo 1 μM) were injected at the indicated times. The mean±S.E.M. is representative of three independent experiments carried out in quadruplicate. (**e**) The total cellular ATP levels in cells incubated in normoxia for 48 h were standardized to protein content for each condition. The mean±S.E.M. is representative of three independent experiments carried out in quadruplicate. Non significative (n.s.). (**f**) *In vitro* proliferation of *Ampk*^+/+^ (*Ampk*^+/+^) and *Ampk*^−/−^ (*Ampk*^−/−^) MEFs transformed by Ras^V12^ incubated in normoxia (Nx) or in hypoxia 1% O_2_ (Hx) up to 96 h. The mean±S.E.M. is representative of three independent experiments carried out in duplicate. (**g**) Clonal growth in normoxia (Nx) or hypoxia 1% O_2_ (Hx) of *Ampk*^+/+^ (*Ampk*^+/+^) and *Ampk*^−/−^ (*Ampk*^−/^) MEFs transformed by Ras^V12^ for 8 days before staining and visualization of the colonies.

Wild-type murine embryonic fibroblasts (MEFs) (*Ampk^+/+^*) or deficient for the expression of both the AMPKα1 and AMPKα2 catalytic subunits (*Ampk^−/−^*) were transformed with oncogenic Ras^V12^ (Figure 1a). Transformation did not change the ability of AMPK to be activated in the absence of glucose and to activate Acetyl-CoA carboxylase (P-ACC), one of the main targets of AMPK (Figure 1b). We sought to determine whether the lack of functional AMPK affected metabolism, ATP levels and cell proliferation. The oxygen consumption rate (OCR) showed no significant difference between *Ampk^+/+^* and *Ampk^−/−^* MEFs (Figure 1c). Oligomycin (Oligo), an inhibitor of the F0F1-ATP synthase, reduced the OCR to a similarly to that of AMPK in *Ampk^+/+^* or *Ampk^−/−^* MEFs suggesting that absence of AMPK did not modify the amount of ATP produced by mitochondrial respiration. The extracellular acidification rate (ECAR), the index of lactic acid export and thus glycolysis, was also identical in cells with and without functional AMPK, in the absence or presence of glucose (Figure 1d). Inhibition of mitochondrial ATP synthesis by oligomycin was responsible for a rapid shift toward glycolysis metabolism, independently of AMPK. Similarly, no significant difference in the ATP level was observed in optimal conditions of cell growth (Figure 1e). Finally, *Ampk^+/+^* and *Ampk^−/−^* MEFs proliferated *in vitro* in normoxia or hypoxia in the presence of 25mM glucose (Figure 1f and 1g). However, *Ampk*^−/−^ MEFs proliferated slightly less rapidly in hypoxia. Taken together, these results suggest that AMPK is dispensable under conditions of plentiful nutrients, either in normoxia or hypoxia.

### Combined inhibition of MCT1 and MCT4 blocked the export of lactate and inhibited glycolysis independently of AMPK

**Figure 2:**
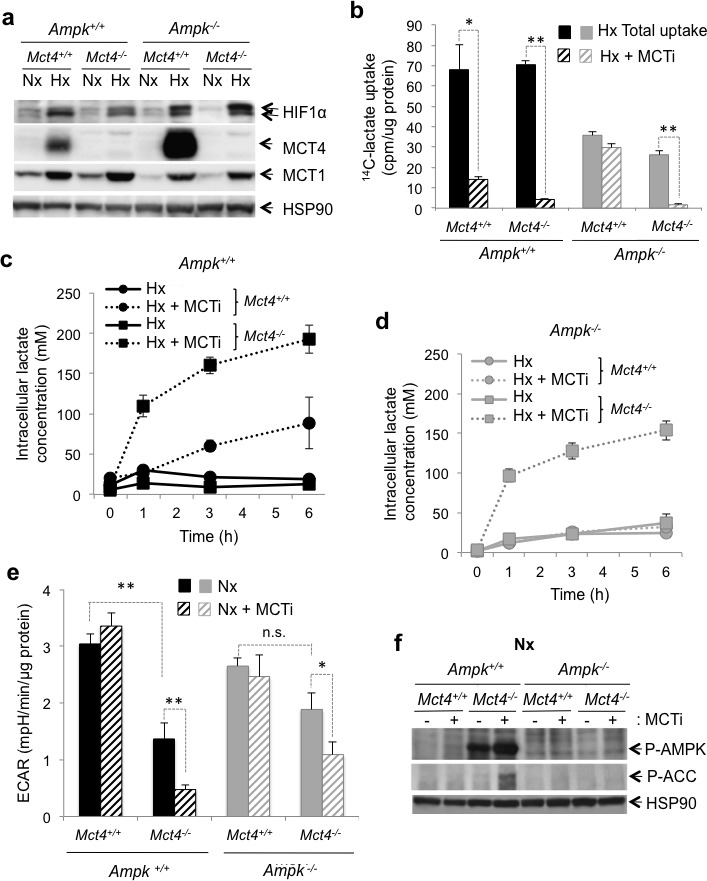
The MCT1 pharmacological inhibitor (MCTi) reduced lactate transport and the glycolytic rate in *Ampk^+/+^* and *Ampk^−/−^* MEFs (**a**) Cell lysates of *Ampk*^+/+^ (*Ampk*^+/+^) and *Ampk*^−/−^ MEFs (*Ampk*^−/−^) expressing (*Mct4*^+/+^) or not (*Mct4*^−/−^) MCT4 incubated in normoxia (Nx) or hypoxia 1% O_2_ (Hx). Whole-cell lysates were analyzed by immunoblotting for HIF-1α, MCT4, and MCT1. Detection of HSP90 was used as a loading control. (**b**) Lactate uptake in *Ampk*^+/+^ (*Ampk*^+/+^, black bars) and *Ampk*^−/−^ (*Ampk*^−/−^, grey bars) MEFs expressing (*Mct4*^+/+^) or not (*Mct4*^−/−^) MCT4 in the absence (total uptake, filled bars) or presence of 300 nM MCTi (MCTi, hatched bars) in hypoxia 1% O_2_ (Hx) for 48 h. Uptake was conducted as 3-min time-points in duplicate. The mean±S.E.M. is representative of two independent experiments carried out in duplicate. * *p* < 0.005, ** *p* < 0.001. (**c**) Time-course of intracellular lactate concentration in response to glucose addition (25 mM) to *Ampk*^+/+^ (*Ampk*^+/+^) MEF expressing (*Mct4*^+/+^) or not (*Mct4*^−/−^) MCT4 in the presence of DMSO (solid line) or MCTi (300 nM, dotted line) in hypoxia 1% O_2_ (Hx). The mean±S.E.M. is representative of three independent experiments carried out in triplicate. (**d**) Time-course of intracellular lactate concentration in response to glucose addition (25mM) to *Ampk*^−/−^ MEFs expressing (*Mct4*^+/+^) or not (*Mct4*^−/−^) MCT4 in the presence of DMSO (solid line) or MCTi (300 nM, dotted line). The mean±S.E.M. is representative of three independent experiments carried out in triplicate. (**e**) The extracellular acidification rate (ECAR) of *Ampk*^+/+^ (*Ampk*^+/+^, black bars) and *Ampk*^−/−^ MEFs (*Ampk*^−/−^, grey bars) expressing (*Mct4*^+/+^) or not (*Mct4*^−/−^) MCT4 with DMSO (filled bars) or MCTi (300nM, signs in bars) in normoxia (Nx) were evaluated after injection of glucose (10 mM) with a Seahorse XF. The mean±S.E.M. is representative of three independent experiments carried out in quadruplicate. * *p* < 0.005, ** *p* < 0.001. (**f**) Cell lysates of *Ampk*^+/+^ (*Ampk*^+/+^) and *Ampk*^−/−^ (*Ampk*^−/−^) MEFs expressing (*Mct4*^+/+^) or not (*Mct4*^−/−^) MCT4 in normoxia (Nx) in the absence (−) or presence (+) of 300nM MCTi were analyzed by immunoblotting for Phospho-AMPK (P-AMPK), and Phospho-ACC (P-ACC). Detection of HSP90 was used as a loading control.

To inhibit glycolysis, we blocked the output of cellular lactate by modulating the expression and/or activity of MCT1 and 4. MCT1 was expressed in normoxia (Figure 2a). While MCT1 is not a transcriptional target of HIF-1, we observed that its expression was three-fold higher (3.3±0.5) in hypoxia whereas the level of expression of the mRNA was not modified (data not shown). However, protein expression of MCT4 was undetectable in normoxia, but was strongly induced in hypoxia (Figure 2a). MCT4 expression was higher in *Ampk^−/−^* MEFs than in *Ampk^+/+^* MEFS in hypoxia. Since no specific inhibitor of MCT4 is available, we knocked out the *Mct4* gene (*Mct4^−/−^* ) in *Ampk^+/+^ and Ampk^−/−^* MEFs. Knockout did not alter expression of MCT1 (Figure 2a). Genetic knockout of *Mct4* in *Ampk^+/+^* and *Ampk^−/−^* MEFs did not alter lactate transport in hypoxia (Figure 2b), suggesting that MCT1 could compensate fully for the lack of MCT4 expression. Pharmacological inhibition of MCT1 (MCTi) in cells lacking MCT4 (*Ampk^+/+^ Mct4*^−/−^ and *Ampk^−/−^ Mct4^−/−^* MEFs) abolished lactate transport in hypoxia (Figure 2b and Supplementary Figure 1a) and thus leaded to its intracellular accumulation in *Ampk^+/+^ Mct4^−/−^* MEFs (Figure 2c) and *Ampk^−/−^ Mct4^−/−^* MEFs (Figure 2d and Supplementary Figure 1b). However, *Mct4* knockout reduced the glycolytic flux of *Ampk^+/+^*cells compared to *Ampk^−/−^* MEFs (Figure 2e). Inhibition of MCT1 in *Mct4^−/−^* MEFs dramatically reduced the glycolytic flux in the presence or absence of functional AMPK in normoxia (Figure 2e and Supplementary Figure 1c). Inhibition of glycolysis was not associated with an increase in mitochondrial respiration (Supplementary Figure 1d and 1e). We also observed that consumption of glucose (Supplementary Figure 2a) and lactate secretion in the extracellular medium (Supplementary Figure 2b) were both affected by combined inhibition of MCT1 and MCT4 in *Ampk*^+/+^ ou *Ampk*^−/−^ MEFs, in normoxia and hypoxia, confirming a decrease in the glycolytic flux in response to inhibition of MCTs. Finally, AMPK was activated in response to MCT inhibition in *Ampk^+/+^ Mct4^−/−^* MEFs (Figure 2f and Supplementary Figure 2c), as shown by the active phosphorylation at Thr172 of the AMPKα subunit (P-AMPK) and of ACC. Colon adenocarcinoma LS174 cells were used to verify that activation was not only specific to MEFs but also found in a model of glycolytic tumour cells. We demonstrated that AMPK activation was faster in LS174 cells in response to inhibition of MCTs (Supplementary Figure 2d). AMPK and ACC phosphorylation occured after 15min, was maintained over time and required combined inhibition of MCT1 and MCT4 (Supplementary Figure 2e). Activation was probably due to the stress in energy generated by inhibition of glycolysis, as recently reported in LS174 cells [17].

Although slight differences were observed between *Ampk*^+/+^ and *Ampk*^−/−^ MEFs (Figure 2a, 2b and 2e), together these results show that inhibition of MCTs lead to intracellular accumulation of lactate and a dramatic reduction in the glycolytic flux in MEFs with and without functional AMPK.

### AMPK is not required for maintenance of a viable ATP level following blockade in glycolysis

**Figure 3:**
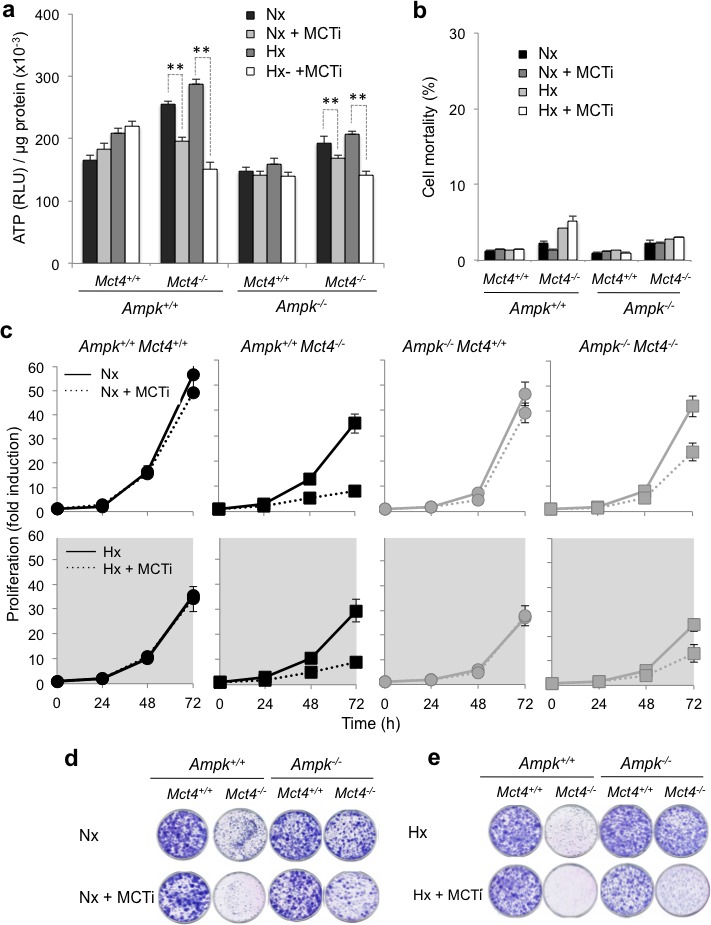
Pharmacological inhibition of MCT1 (MCTi) in combination with knockout of *Mct4* (*Ampk^+/+^ Mct4^−/−^* and *Ampk^−/−^ Mct4^−/−^* MEFs) decreased the ATP level and the proliferation independently of the presence or absence of AMPK but did not alter cellular viability (**a**) Total cellular ATP level in *Ampk*^+/+^ (*Ampk*^+/+^) and *Ampk*^−/−^ (*Ampk*^−/−^) MEFs expressing (*Mct4*^+/+^) or not (*Mct4*^−/−^) MCT4 incubated in normoxia (Nx) or in hypoxia 1% O_2_ (Hx) for 48 h in the absence (DMSO) or presence (+ MCTi, 300 nM) of MCTi. ATP levels were measured in whole-cell lysates and standardized to the cell protein content for each condition. The mean±S.E.M. is representative of three independent experiments carried out in triplicate. The mean±S.E.M. is representative of three independent experiments carried out in quadruplicate. ** *p* < 0.001. (**b**) *Ampk*^+/+^ (*Ampk*^+/+^) and *Ampk*^−/−^ MEFs (*Ampk*^−/−^) expressing (*Mct4*^+/+^) or not (*Mct4*^−/−^) MCT4 incubated in normoxia (Nx) or hypoxia 1% O_2_ (Hx) for 48h in the absence or the presence (+ MCTi, 300 nM) of MCT inhibitor. Cell mortality (%) was evaluated by Trypan blue exclusion. The mean±S.E.M. is representative of three independent experiments carried out in duplicate. (**c**) *In vitro* exponential growth of *Ampk*^+/+^ (*Ampk*^+/+^) and *Ampk*^−/−^ MEFs (*Ampk*^−/−^) expressing (*Mct4*^+/+^) or not (*Mct4*^−/−^) MCT4 incubated up to 72 h in normoxia (Nx – top panel) or hypoxia 1% O_2_ (Hx – bottom panel) in the absence or the presence of the MCT inhibitor (+ MCTi, 300 nM). The mean±S.E.M. is representative of three independent experiments carried out in duplicate. (**d**) Clonal growth in normoxia (Nx) of *Ampk*^+/+^ (*Ampk*^+/+^) and *Ampk*^−/−^ (*Ampk*^−/−^) MEFs expressing (*Mct4*^+/+^) or not (*Mct4*^−/−^) MCT4, in the absence or presence of the MCT inhibitor (+ MCTi, 300 nM) for 8 days before staining and visualization of the colonies. (**e**) Clonal growth in hypoxia 1% O_2_ (Hx) of *Ampk*^+/+^ (*Ampk*^+/+^) and *Ampk*^−/−^ (*Ampk*^−/−^) MEFs expressing (*Mct4*^+/+^) or not (*Mct4*^−/−^) MCT4, in the absence or presence of the MCT inhibitor (+ MCTi) for 8 days before staining for visualization of the colonies.

We then looked at the impact of blocking glycolysis on ATP levels in the absence of AMPK. We analyzed the levels of ATP in *Ampk^+/+^* and *Ampk^−/−^* MEFs, in the presence (*Mct4^+/+^*) or the absence (*Mct4^−/−^*) of MCT4, and after addition or not of MCTi (Figure 3a and Supplementary Figure 3a). In control MEFs (*Ampk^+/+^ Mct4^+/+^* ), we observed that the ATP level increased slightly and reproducibly, although not significantly, after 48h of MCTi treatment in normoxia. As we previously observed, using different cell lines [45, 46], 48h of hypoxia significantly increased ATP levels (25%±5%) compare to the levels in normoxia. Inhibition of MCT1 also showed a slight tendency to increase the ATP level in cells expressing MCT4 in hypoxia. Knockout of *Mct4* in *Ampk^+/+^* MEFs increased basal levels of ATP in normoxia and hypoxia, probably due to inhibition of proliferation. MCT1 inhibition in *Ampk^+/+^ Mct4^−/−^* MEFs reduced the ATP level in normoxia and hypoxia by about 23%±3% and 47%±3%, respectively. The most important effect of MCT inhibition in hypoxia was due mostly to the metabolic shift toward glycolysis, which made the cells more dependent on this pathway for ATP production [15-17]. The basal level of ATP of *Ampk^−/−^* MEFs in normoxia was similar to that of *Ampk^+/+^* MEFs but not modified by inhibiting MCT1 or by hypoxia. Similar to *Ampk^+/+^ Mct4^−/−^* MEFs, *Mct4* knockout in *Ampk^−/−^* MEFs (*Ampk^−/−^ Mct4^−/−^*) increased the basal level of ATP. However, cells were more sensititive to MCT1 inhibition. Indeed, the level of ATP was reduced by about 16%±3% in normoxia and 28%±3% in hypoxia for the first clone (Figure 3a) and 14%±5% in normoxia and 29%±3% in hypoxia for the second independent clone (Supplementary Figure 3a). In the presence of MCTi in normoxia or hypoxia, decreases in ATP levels did not correlate with a significant increase in cell death in *Ampk^+/+^ Mct4^−/−^* or *Ampk^−/−^ Mct4^−/−^* MEFs over a short period of time (8 and 2h, data not shown) or 48h (Figure 3b and Supplementary Figure 3b). We examined the ability of *Ampk^−/−^* (Figure 3c, right panels) to grow in the presence or the absence of MCT4 in normoxia (top panels) or hypoxia (Figure 3c, bottom panels) compared to *Ampk^+/+^* MEFs (Figure 3c, left panels). In normoxia, the addition of MCTi did not alter the proliferation of MEFs expressing MCT4 (*Ampk^+/+^ Mct4^+/+^* et *Ampk^−/−^ Mct4^+/+^*) (Figure 3c). When *Mct4* was knocked out, inhibition of MCT1 greatly reduced proliferation at 48h (*Ampk^+/+^ Mct4^−/−^* ou *Ampk^−/−^ Mct4^−/−^* ) but less in MEFs that did not express functional AMPK. At 72h, both inhibition of MCT1 and knockout of MCT4 led to a stronger inhibition of proliferation in *Ampk^+/+^* in normoxia (76%±0.6%) than that in *Ampk^−/−^* MEFs (32%±3%). In hypoxia, *Ampk^+/+^* and *Ampk^−/−^* MEFs proliferated more slowly than that in normoxia and MCT1 inhibition had no significant effect on these cells (Figure 3c). In contrast, MCTi inhibited proliferation of *Mct4-* deficient cells, in a manner similar to that of *Ampk^+/+^ Mct4^−/−^* MEFs (75%±0.6%) and *Ampk^+/+^ Mct4^−/−^* MEFs (62%±0.8%) (Figure 3c and Supplementary Figure 3c). Cell growth and/or mortality over a long period of time in normoxia (Figure 3d and Supplementary Figure 3d) and in hypoxia (Figure 3e and Supplementary Figure 3e) were determined by the clonogenicity assay. Proliferation was strongly reduced in *Ampk^+/+^ Mct4^−/−^* or *Ampk^−/−^ Mct4^+/+^* MEFs in the presence of MCTi in both conditions.

Although small differences between *Ampk*^+/+^ and *Ampk*^−/−^ MEFs were found, all these results suggest that after blockade of lactate export and inhibition of glycolysis, MEFs did not require the presence of AMPK to maintain a viable level of ATP *in vitro*.

### AMPK did not confer a survival benefit following the combined blockade of glycolysis and respiration, but induced resistance to glucose deprivation

**Figure 4:**
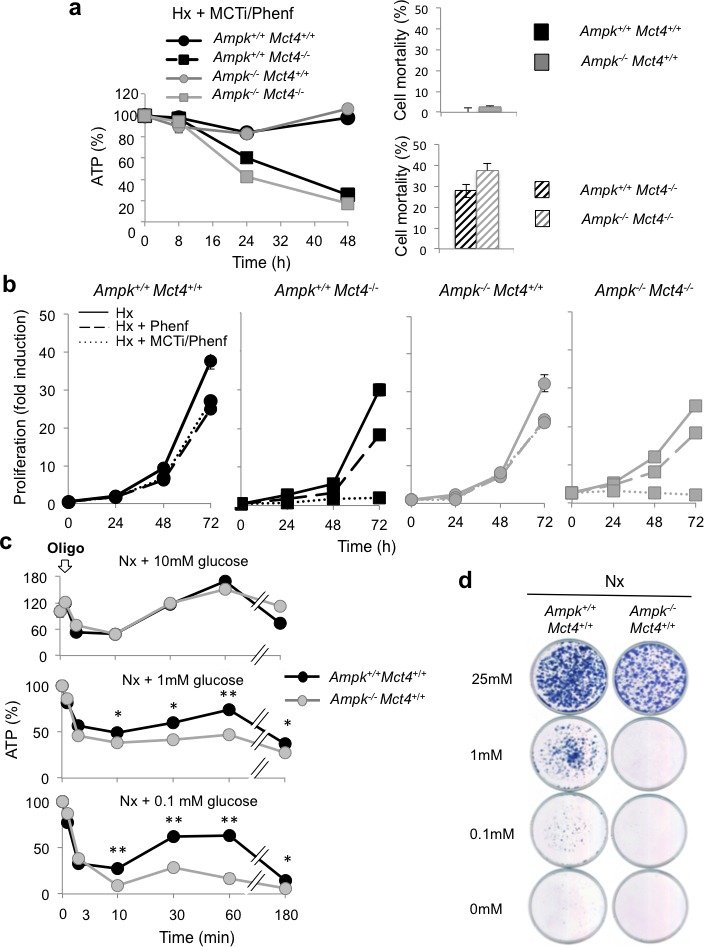
AMPK is not sufficient to guaranty viability in response to inhibition of MCT1 (MCTi) and MCT4 (*Mct4^−/−^*) inhibition combined with phenformin (Phenf), but is important to promote cell survival following a nutritive stress (**a**) Left panel - Total cellular ATP level in *Ampk*^+/+^ (*Ampk*^+/+^) and *Ampk*^−/−^ (*Ampk*^−/−^) MEFs expressing (*Mct4*^+/+^) or not (*Mct4*^−/−^) MCT4 incubated in hypoxia 1% O_2_ (Hx) for up to 48 h in the presence of a combination with MCTi (300 nM) and phenformine (50 μM) (+ MCTi/Phenf), standardized to the protein content for each condition and normalized to initial cellular ATP level (%). The mean±S.E.M. is representative of three independent experiments carried out in quadruplicate. Right panel –*Ampk*^+/+^ (*Ampk*^+/+^) and *Ampk*^−/−^ (*Ampk*^−/−^) MEFs in the presence (black signs in bars) or absence (grey signs in bars) of *Mct4* (± *Mct4*) were incubated in normoxia (Nx) for 48 h with the MCTi/Phenf combination, 300 nM and 50μM respectively. Cell mortality (%) was evaluated with the automatic cell counter ADAM. The mean±S.E.M. is representative of three independent experiments carried out in triplicate. (**b**) *In vitro* exponential growth of *Ampk*^+/+^ (*Ampk*^+/+^) and *Ampk*^−/−^ MEFs (*Ampk*^−/−^) expressing (*Mct4*^+/+^) or not (*Mct4*^−/−^) MCT4 incubated for up to 72 h in hypoxia 1% O_2_ (Hx) in the absence or presence of phenformine (+ Phenf) or presence of the MCTi/Phenf combination (+ MCTi/Phenf), 300 nM and 50 μM respectively. The mean±S.E.M. is representative of three independent experiments carried out in duplicate. (**c**) Total cellular ATP level in *Ampk*^+/+^ (*Ampk*^+/+^) and *Ampk*^−/−^ (*Ampk*^−/−^) MEFs expressing MCT4 (*Mct4*^+/+^) incubated in normoxia (Nx) in 10-, 1- and 0.1-mM glucose, in the presence of oligomycine (Oligo, 1 μM) for up to 180min, standardized to the cell protein content for each condition and normalized to initial cellular ATP level (%). The mean±S.E.M. is representative of three independent experiments carried out in triplicate. The mean±S.E.M. is representative of two independent experiments carried out in quadruplicate. * *p* < 0.005, ** *p* < 0.001. (**d**) Clonal growth in normoxia (Nx) of *Ampk*^+/+^ (*Ampk*^+/+^) and *Ampk*^−/−^ (*Ampk*^−/−^) MEFs expressing MCT4 (*Mct4*^+/+^) in 10-, 1- and 0.1-mM glucose for 3 days then incubated for 10 days in 25 mM glucose before staining for visualization of the colonies.

*Ampk^+/+^* and *Ampk^−/−^* MEFs expressing MCT4 (*Mct4^+/+^*) or not (*Mct4^−/−^*) were treated in hypoxia with MCTi combined with phenformin (MCTi/Phenf) an inhibitor of mitochondrial respiratory chain complex 1. Combination of MCTi/Phenf did not alter ATP levels in MEF expressing MCT4 (*Ampk^+/+^ Mct4^+/+^* et *Ampk^−/−^ Mct4^+/+^* MEFs) (Figure 4a, left panel). In *Ampk^+/+^ Mct4^−/−^* MEFs, while phenformin alone did not affect the level of ATP (Supplementary Figure 4a) the combination of MCTi/Phenf gradually reduced the ATP pool from 97%±1, 60%±1 and 25%±3 of its initial level after 8, 24 and 48h of treatment, respectively (Figure 4a, left panel). A reduction in the ATP level in the *Ampk^−/−^ Mct4*^−/−^ MEFs was similar to that of *Ampk^+/+^ Mct4*^−/−^ MEFs. No difference in mortality was observed between *Ampk^+/+^ Mct4^+/+^* or *Ampk^−/−^ Mct4^+/+^* MEFs (Figure 4a, right panel). However, for *Ampk^+/+^ Mct4*^−/−^ and *Ampk^−/−^ Mct4*^−/−^ MEFs 27%±3% and 36%±4% cell mortality was observed*,* respectively. Similar results were obtained when ATP was collapsed with the MCTi/Oligo combination (Supplementary Figure 4b). Phenformin slowed cell proliferation in a similar manner: 34%±1% (*Ampk^+/+^ Mct4^+/+^*), 39%±4% (*Ampk^+/+^ Mct4*^−/−^), 30%±6% (*Ampk^−/−^ Mct4^+/+^*) and 28%±3.8% (*Ampk^−/−^ Mct4*^−/−^) (Figure 4b) without affecting cell viability (data not shown). Combination of MCTi/Phenf decreased slightly the cell number over 72 h in a similar manner in *Ampk^+/+^ Mct4^+/+^* (28%±1) and *Ampk^−/−^ Mct4^+/+^* (33%±2) MEFs (Figure 4b). However, the cell number was significantly reduced by 94%±2% in *Ampk^+/+^ Mct4*^−/−^ MEFs and by 91%±2% in *Ampk^+/+^ Mct4*^−/−^ MEFs after 72 h of MCTi/Phenf treatment. The same effect was observed after 8 days of growth using the clonogenicity assay (Supplementary Figure 4c).

Taken together these results showed that MCT4 knockout sensitized MEFs to the MCTi/Phenf combination, which reduced substantially the ATP level, viability and proliferation. Knockout of AMPK was neither beneficial nor unfavourable to these parameters.

The question then was whether AMPK provides an advantage in cell survival in response to a rapid decrease in the level of ATP. The MCTi/Phenf combination impacted on the ATP levels after 24h of treatment (Figure 4a). As oligomycin rapidly blocked production of ATP in contrast to phenformin, it was added in the presence of decreasing concentrations of glucose (10 mM, 1mM or 0.1 mM) (Figure 4c). In the presence of 10mM glucose oligomycin dropped the level of ATP (49%±6%) in 10min either in the *Ampk^+/+^* or *Ampk^−/−^* MEFs,. This level was gradually restored, and even reached values above the basal level of ATP 1h after treatment (Figure 4c, top panel). When oligomycin was added to medium containing 1mM glucose, ATP levels dropped rapidly to similar levels to those in the presence of 10 mM glucose (49%±3% and 38%±2% for *Ampk^+/+^* or *Ampk^−/−^* MEFs, respectively) (Figure 4c, middle panel). However, none of these two populations of MEFs completely restored their basal level of ATP. When oligomycin was added to medium containing 0.1mM glucose, a significant difference was observed between levels of ATP for *Ampk^+/+^* compared to *Ampk^−/−^* MEFs, 28%±3% versus 9%±2%, respectively (Figure 4c, bottom panel). This difference was amplified after 1h of treatment as *Ampk^+/+^* MEFs had an ATP level of 63%±0.5% compared to *Ampk^−/−^* MEFs (28%±3%). In the absence of 10 mM glucose±2-deoxy-glucose, oligomycin collapsed ATP production, independently of the AMPK activity (Supplementary Figure 4d and 4e). Little difference in cell number was observed between *Ampk^+/+^* and *Ampk^−/−^* MEFs in the presence of 25 mM glucose (Figure 4d). However, in the presence of 0.1 or 1mM glucose, proliferation of *Ampk^+/+^* MEFs slowed while a decrease in the glucose concentration dramatically impacted cell viability of *Ampk^−/−^* MEFs.

Taken together these results suggest that AMPK is not required to maintain a viable level of ATP in response to inhibition of glycolysis *via* blockade of MCTs or metabolic shock (MCTi/Phenf). The only differences observed were obtained under conditions of low glucose concentrations (0.1 and 1 mM).

### Knockout of AMPK or MCT4 or both strongly impact on the tumourigenicity

**Figure 5:**
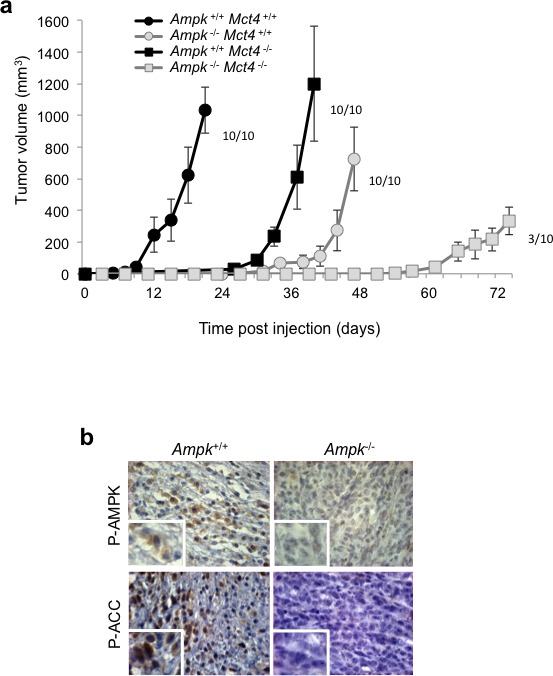
Dual knockout of *Ampk* (*Ampk^−/−^*) and *Mct4* (*Mct4^−/−^*) dramatically decreased xenograft tumour development (**a**) *In vivo* xenograft assays were performed by injecting s.c. into the back of athymic nude mice 1×10^6^ viable and individual tumour *Ampk*^+/+^ (*Ampk*^+/+^) or *Ampk*^−/−^ (*Ampk*^−/−^) MEFs expressing (*Mct4*^+/+^) or not (*Mct4*^−/−^) MCT4. Xenograft growth was determined by measuring the tumour volume. *In vivo* experiments were repeated twice. Five mice were studied per condition. (**b**) Immunohistological confirmation of the expression of Phospho-AMPK (P-AMPK) and Phospho-ACC (P-ACC) in the corresponding *Ampk*^+/+^ and *Ampk*^−/−^ tumour xenografts (Magnification: 20×).

We then investigated the behaviour of these cells in a tumour context. *Ampk^+/+^ Mct4*^+/+^ control MEFs showed rapid tumour growth, up to 200mm^3^ after 12 days (±3) and greater than 1000mm^3^ after 21 days of injection (Figure 5a). We observed a high latency in tumour growth with *Ampk*^−/−^*Mct4^+/+^* MEFs as the tumours reached 200mm^3^ after 44 days (±6) post-injection. However, after this period, the rate of tumour growth was identical for *Ampk^+/+^* and *Ampk*^−/−^ MEFs (Figure 5a). Moreover, *Ampk^+/+^ Mct4*^−/−^ MEF showed slowed tumourigenicity but not tumour growth as *Ampk^+/+^ Mct4*^−/−^ MEFs exhibited a tumour volume of 200mm^3^ to 1000mm^3^ in 33 days (±6) and 40 days (±6), respectively (Figure 5a). *Ampk*^−/−^ *Mct4*^−/−^ MEF-derived tumours grew much slower (Figure 5a). The absence of AMPK activity in MEF-derived tumuors was confirmed (Figure 5b).

These results clearly show that independently AMPK and MCT4 impact on tumour development but when taken together the *in vivo* consequence is reinforced.

## DISCUSSION

AMPK is widely recognized as the guardian of the balance in energy and as a critical regulator of intracellular ATP level. However, in the present study, we showed for the first time, that AMPK was dispensable for maintenance of the ATP level and viability of fibroblasts when glycolysis was limited by MCT blockade. However, we confirmed that *Ampk^−/−^* MEFs were more sensitive to glucose deprivation or to acute energy stress than their wild type counterpart. While the double knockout of AMPK and MCT4 (*Ampk^−/−^ Mct4^−/−^* ) did not drastically change the ATP level or the characteristics of growth *in vitro*, it showed a marked decrease in the tumour incidence. These results lead us to ask the following questions: (i) What are the differences, from an energy point of view, between inhibiting glycolysis by restricting lactic acid export versus decreasing availability of glucose? (ii) Is AMPK a tumour suppressor or an oncogene in our model? (iii) Why did the double knockout of AMPK and MCT4 reduce substantially tumourigenicity while no phenotypic differences were found *in vitro*?

Our approach to reducing tumour growth by blocking the last step of fermentative glycolysis (export of lactic acid) [15] was recently confirmed in and extended to other cancer cell lines [17, 25, 26, 47]. Although blockade of glycolysis was capable of restricting proliferation, intracellular ATP levels and cell viability were maintained due to re-activation of OXPHOS. Indeed discontinuation of MCTi treatment restored tumour growth indicating a cytostatic effect [15, 8]. The aim of this study was to test the hypothesis that combining inhibition of glycolysis with suppression of AMPK should induce acute tumour cell death from ‘ATP crisis’. In the absence of a specific inhibitor of AMPK, we used Ras-transformed MEFs knocked out for the two isoforms of AMPK, α1 and α 2 [44].

Differences in blockade in energy. We showed that pharmacological inhibition of MCT1 in *Mct4*−/− MEFs in normoxia rapidly led to inhibition of glycolysis, which induced a stress in energy as observed from the activation of AMPK in *Ampk*^+/+^ MEFs (Figure 2f). In this circumstance, deletion of AMPK did not affect either ATP levels (Figure 3a) or the rate of proliferation (Figure 3c) in response to energy stress suggesting that MEFs adjust their energetic homeostasis independently of AMPK. However, a beneficial effect of AMPK in cell survival was evident when MEFs were subjected to limiting concentrations of glucose. Why such a difference? Our main hypothesis to explain this phenomenon lies in the inhibitory effect of pHi on protein synthesis and proliferation. Indeed, inhibition of MCTs is associated with intracellular acidification [15, 17]. However, intracellular acidosis, in addition to reducing the glycolytic flux through the inhibition of hexokinase or phosphofructokinase [48-50] leads to inhibition of mTORC [51-53], one of the most energy-demanding anabolic pathways [54]. Therefore, we proposed that intracellular acidification resulting from our anti-MCT strategy mimicked the action of AMPK under conditions of energy stress: inhibition of mTORC1 and presumably other anabolic pathways. Two recent studies showing that mTORC1 may be inhibited in response to energy stress through a mechanism that does not involve AMPK [55, 56], support our hypothesis. In contrast, when the glucose concentration was reduced from 10 to 1mM, a significant decrease in the rate of glycolysis was observed. Moreover, suppression of glycolysis was reached at 0.3mM glucose. Under these conditions cellular growth stopped and MEFs maintained their ATP levels through OXPHOS using glutamine and residual glucose. Thereby, AMPK facilitated cell survival by supporting energy reprogramming through blockade of anabolic pathways. These more pathophysiological conditions of energy stress obtain by a gradual deficiency in glucose allow a better understanding of the beneficial effect on survival of cells expressing AMPK.

It is now well established that glycolysis is closely linked to cell proliferation [2, 57]. Differentiated cells and cells with a low proliferative rate depend almost exclusively on cellular respiration to produce cellular ATP. To counteract this metabolic reprogramming, we combined inhibition of mitochondrial respiration (oligomycin or phenformin) with inhibition of glycolysis. The conclusion regarding the benefit of AMPK in these conditions seems to depend on the intensity/kinetics of the imposed “metabolic block”. Indeed, the MCTi/Phenf combination, by gradually decreasing the ATP pool, affected similarly *Ampk*^+/+^ and *Ampk*^−/−^ MEFs (Figure 4a). However, the MCTi/Oligo combination, which affected more rapidly ATP levels than the previous combination, had a stronger effect on ATP levels in *Ampk*^−/−^ MEFs after 24 or 48 h of treatment (Supplementary Figure 4b). In conclusion, the apparent role of AMPK in regulating the level of ATP is mainly highlighted when glycolysis is inhibited by a gradual reduction in the concentration of its critical substrate, glucose (Figure 4c).

AMPK and tumour development. The role of AMPK in cancer is controversial [58]. AMPK is both a tumour suppressor, responsible for a cytostatic effect when activated, and a protector of tumour cells, allowing them to survive in a hostile environment for extended periods of time, which occurrs during development of solid tumours [59, 60]. In our study, we observed a strong delay in tumour establishment of *Ampk*^−/−^ MEFs compared to *Ampk*^+/+^ MEFs. This tumour growth delay, also described by Laderoute *et al.* [44], confered to AMPK a protective or a pro-tumoural role. However, when tumour growth was initiated, the rates of tumour growth of *Ampk*^+/+^ and *Ampk*^−/−^ MEFs were similar, which is not in favour of an “oncogene” function. We propose that the delay observed at the time of tumour establishment would reflect a much higher sensitivity of *Ampk*^−/−^ MEFs to the poorly vascularised microenvironment, which is low in oxygen and glucose. Results reported in Figure 4d reinforce this hypothesis. Indeed, they showed that *Ampk*^−/−^ MEFs are particularly sensitive to a decrease in glucose availability, an omnipresent feature during the initiation phase of solid tumour establishment. Moreover, Faubert *et al.* showed accelerated development of lymphomas in transgenic *Ampkα1* knockout mice (single subunit expressed in B cells) overexpressing c-Myc [61]. This result is consistent with the normal tumour growth rate observed for *Ampk*^−/−^ MEFs after tumour establishment. However, the question of the role of AMPK in initiating solid tumours remains open. Indeed, both models developped by Faubert or our selves failed to clearly define the positive or negative effect of AMPK during establishment of solid tumour. However, we provided additional evidence that AMPK is not a key oncogene in tumour growth after their establishment. In contrast to Li *et al.* [62] or Zhou *et al.* in [63] who studied ovarian and prostate cancer, respectively, loss of AMPK did not promote tumour growth in Ras-transformed MEFs.

MCT4 and AMPK, a dynamic duo for tumour growth. In contrast to studies that have largely focused on MCT1 [25, 47, 64], our team has developed the notion that dual blockade of MCT1 and MCT4 is critical to arrest glycolytic tumour growth [15, 17, 26]. Here we showed that *Mct4* knockout alone severely affected tumour establishment of both *Ampk*^+/+^ and *Ampk*^−/−^ MEFs in subcutaneous xenografts. However, it did not affect the tumour growth rate suggesting that in this cellular setting MCT1 did not ensure sufficient export of lactate on its own. It also established that the function “lactate transport” is crucial when establishing solid tumours, at least in the model of murine tumour xenografts. These results are consistent with those obtained with the Ras-transformed fibroblast CCL39-derived mutants impaired in mitochondrial respiration [65] and expressing MCT1 and not MCT4 [15]. These respiratory-deficient CCL39-derived mutants showed reduced tumour incidence (20%), whereas ectopic expression of MCT4 was sufficient to restore full tumourigenicity (100%) [15]. Forced expression of MCT4 in these cells induced a more alkaline cytoplasmic pH with concomitant activation of glycolysis [66].

Our study showed that *Mct4* knockout in a *Ampk*^−/−^ context severely affected both tumour establishment and tumourigenicity, demonstrating an important role for AMPK and MCT4 in tumour development. How do we explain the effect of *Ampk* /*Mct4* double-knockout on tumourigenicity when we observed no or little phenotypic differences of these cells *in vitro*, compared to single *Ampk*^−/−^ MEF knockout? Could MCT4 be involved specifically in proliferation in a 3D context, in angiogenesis, or in metabolic interactions with the tumour microenvironment? A recent study demonstrated that *Mct4* knockdown by shRNA reduced both the stem cell population (CD133^+^) and the glioblastoma tumour growth by an unknown mechanism, independently of its function of lactate transporter [67]. Such an unknown mechanism could be involved in the absence of development of *Mct4^−/−^* tumours. Moreover, the absence of AMPK could also amplify this phenomenum.

Molecules targeting MCT4 are currently under development, and our *in vivo* results suggest that these molecules could have some therapeutic interest in the context of restricted AMPK activity. Unfortunately, currently there is no specific inhibitor of AMPK. However, as LKB1, the upstream kinase of AMPK, has been found to be mutated in several tumour types [68], one could consider that these cancers have a deficiency in the AMPK activity. Targeting MCT4 in these tumours is an attractive therapeutic opportunity worth investigation.

Finally, this study revealed that the bioenergetic plasticity in regulation of glycolysis and OXPHOS could occur in AMPK-null cells. We propose that intracellular acidification resulting from lactic acid sequestration mimicks AMPK by blocking mTORC1, a major ATP consuming pathway, therefore preventing killing by ‘ATP crisis’. In line with cell killing we are left with the option of targeting MCTs with acute treatment with the anti-diabetic drug phenformin [17, 26, 27]. Paradoxically phenformin is an activator of AMPK but, as shown here, it kills cells treated with MCTi independently of the AMPK status and *via* inhibition of mitochondrial complex I.

## MATERIALS AND METHODS

### Cell culture

*Wild-type* (*Ampk*^+/+^) and double knockout for *Ampk*α*1* and *Ampk*α*2* (*Ampk^−/−^*) murine embryonic fibroblasts (MEFs), were previously described [44] and were kindly provided by Dr B. Viollet (Institut Cochin, Paris, France). They were transformed (Retroviruses – System Phoenix) with the oncogene Ras^V12^ (plasmid #1768 from Addgene) following the Retrovirus Production and Infection from the Rockefeller University protocols. MEFs and the human colon carcinoma LS174 cell line were grown in Dulbecco's modified Eagle's medium (DMEM) (Gibco-BRL) supplemented with 10% foetal bovine serum (FBS). LS174-*Mct4^−/−^* were previously described [15]. Penicillin G (50 U/ml) and streptomycin sulfate (50μg/ml) were added. When experiments were performed with medium containing 0.1 or 1mM glucose, medium was changed every 24h to minimize variations in the glucose concentration in the medium during the time of the experiment.

For hypoxic cultures the Bug-BoxTM anaerobic workstation (Ruskinn Technology Biotrace International Plc, Bridgend, UK) was set at 1% or 0.1% oxygen, 94% or 94.9% nitrogen, respectively and 5% carbon dioxide was used.

### Nude mice tumourigenicity and immunohistochemistry

This study was carried out in strict accordance with the recommendations in the Guide for the Care and Use of Laboratory Animals of Centre National de la Recherche Scientifique (CNRS). All efforts were made to minimize suffering. Our experiments were approved by the “Comité national institutionnel d’éthique pour l'animal de laboratoire (CIEPAL)”. The project is registered under the reference: NCE/−165.

ZFN-mediated gene knockout of the *mct4* gene, Colony-forming assay, Proliferation and Cell survival Assay, Immunoblot analysis, Metabolic measurements, ATP determination, Glucose consumption and Lactate measurement. The corresponding methodology is given in the supplemental data.

### Statistics

All values are the means ± SD of the indicate number of determinations (n) and significant differences are based on the Student's *t*-test and *P* values indicated (*P < 0.05, **P < 0.01).

## SUPPLEMENTARY MATERIAL FIGURES


