# Supplementary material for: AMP-activated protein kinase is dispensable for maintaining ATP levels and for survival following inhibition of glycolysis, but promotes tumour engraftment of Ras-transformed fibroblasts
Source: Oncotarget. 2015 Mar 30;6(14):11833–47. doi: 10.18632/oncotarget.3738 (PMC4494908; doi:10.18632/oncotarget.3738)
Supplement: Supplementary file 1 [file oncotarget-06-11833-s001.pdf]

AMP Kinase is dispensable for maintaining ATP levels and for survival following inhibition of glycolysis, but promotes tumour engraftment of ras-transformed fibroblasts

Supplementary Material

ZFN-mediated gene knockout of mct4 gene

Ampk$^{+/+}$ and *Ampk*$^{-/-}$ MEFs were transiently transfected twice in a week interval with a plasmid expressing GFP (0.5µg), and with the two plasmids (10µg each), expressing the zinc finger nuclease (ZFN) designed by Sigma-Aldrich (CompoZr Custom ZFN), and targeting the 3rd transmembrane segment of murine *Mct4*. GFP positive transfected cells were sorted out by flow cytometry (FACSAria, BD Biosciences). Only clones with double allele mutations were selected phenotypically, confirmed by DNA sequencing, and further analysis.

Clonogenicity growth assay

5x10^{3} cells were seeded onto 60mm dishes. 24h later, medium was replaced with regular medium, for 8 days of growth in the presence or not of MCTi (300nM), Phenformin (50µM), or both under normoxic or hypoxic (1% O_2) conditions. Dishes were then stained with Giemsa (Fluka).

Proliferation Assay

5x10^{4} cells were seeded onto 35mm dishes. 24h after seeding, medium was replaced with medium containing the indicated compounds. Cells were detached and counted with Coulter Z1 (Beckman) every 24h during 4 to 5 days. Proliferation units were calculated by dividing the cell number obtained for each day by the one obtained 24h after seeding. Cell counting was done on two independent dishes at each time point and the experiment was repeated at least three times.

Cell Survival Assay

5x10^{4} cells were seeded onto 35mm dishes. At the indicated time, the rate of mortality was assessed using Trypan blue exclusion staining (Sigma, St. Louis, MO).

Immunoblot Analysis

Cells were lysed in 1.5x sodium dodecyl sulfate (SDS)-buffer, and protein concentration was determined using the bicinchoninic acid assay. 40µg of each whole-cell extract were resolved by electrophoresis on SDS-polyacrylamide gels and transferred onto a polyvinylidene difluoride membrane (Millipore). Membranes were blotted with monoclonal antibodies to Phospho-AMPKα (Thr172), Phospho-Acetyl-CoA Carboxylase (Ser79) and to total AMPKα proteins (F6) purchased from Cell Signaling. Rabbit polyclonal antibodies to MCT4 (H-90) and MCT1 (M-45) were purchased from Santa Cruz Biotechnology. The rabbit polyclonal anti-HIF-1α antibody (antiserum 2087) was produced and characterized in our laboratory, and has been reported previously[66]. Antibody to Hsp90 (Abcam) was used as a loading control. The rabbit polyclonal antibody to arrest-defective-1 protein (ARD1) (previously produced and characterised in our laboratory[67]) was also used as loading control. Bands were detected with a horseradish peroxidase antimouse or anti-rabbit antibody (Promega) by ECL (Amersham Biosciences or Millipore).

Metabolic measurements

Glycolytic rates and mitochondrial respiration were assessed by monitoring extracellular acidification and oxygen consumption rates simultaneously in realtime using the Seahorse Biosciences Extracellular Flux Analyzer[68]. Cells were plated at an optimum density (1.5 to $2x10^5$ cells/well) to achieve a confluent layer for the fluorescent probes to detect changes. The experimental procedure involved monitoring cells for oxygen consumption and lactic acid production (extracellular acidification) while injecting metabolic compounds into the medium (glucose 10mM, oligomycin 1µM, FCCP 1µM, rotenone/antimycinA 1µM each, MCTi 300nM). Seahorse Biosciences Assay medium was used during experimentation. This media is an unbuffered DMEM without glucose, pyruvate, and bicarbonate. pHe of the media was adjusted before each use. Data are presented as extracellular acidification rate (ECAR; mpH/min/µg protein) for glycolysis and oxygen consumption rate (OCR; pMolesO_2/min/µg protein). ECAR and OCR were determined at 3min measurement intervals. Protein samples were taken at the end of each experiment to standardize the results. Each assay was performed in quadruplicate at 37°C and representative data from at least three independent experiments are shown.

Determination of intracellular lactate concentration

$5x10^5$ cells were washed twice with PBS and maintained in DMEM without glucose, pyruvate, and serum for 30min with either DMSO (0.1% vol) or MCTi (300nM). At the indicated times (0–6h) after glucose (25mM) and serum addition, cells were washed twice with cold PBS, once with cold water and lysed in cold water (200µL). Lactate concentration was determined by an enzyme based assay using 900µM β-NAD (BioChemika), 175µg/mL lactate dehydrogenase (BioChemika), and 100µg/mL glutamate pyruvate transaminase (Roche) diluted in a sodium carbonate (620mM)-L-glutamate (79mM) buffer adjusted to pH10. Lithium lactate was used as a standard. Measurements were done with a microplate reader after incubation for 30min at 37 °C. Determinations were done in triplicate, and the experiment was repeated at least three times.

Determination of glucose consumption and lactate production

$5x10^5$ cells were seeded onto 60mm dishes and following cell attachment, medium was replaced with 5 ml of medium with DMSO or MCTi (300nM) and cells were incubated in normoxia or hypoxia (1%O_2). 48h later, medium was replaced with a fresh one still containing DMSO/MCTi. Supernatant was collected 1h and 6h later. Then, cells were lysed and protein concentration was determined to normalize lactate production/glucose consumption to protein content. Glucose and lactate concentrations were measured in the supernatant by the Ysi 2300 STAT Plus analyzer (YSI Life Sciences). Rate of lactate production was determined by substracting the amount of lactate in the medium after 6h to the value obtained 1h after addition of the fresh medium. Similarly, rate of glucose consumption was determined by substracting the amount of glucose in the medium after 1h to the value obtained 6h after addition of the fresh medium. Determinations were done in triplicate, and the experiment was repeated two times.

ATP assays

To determine cellular ATP content we utilized a luminescence assay (CellTiter-Glo, Promega). Briefly, 1,000 cells were plated per well in 200µl on a 96 well plate and following cell attachment medium was replaced with 200µl of medium with the indicated compounds. After 48h incubation, the relative ATP content was determined following the manufacturers instructions. An identical plate was always prepared to standardize ATP results to protein content (lysis in 0.1M NaOH, BCA protein assay). For the short lasting experiments (1min to 3h), 5,000 cells were plated per well in 200µl on a 96 well plate. 24h later, cells were washed

twice with PBS, and 100μl of medium with the indicated concentration of glucose was added. 45min later, 100μl of medium with the indicated concentration of glucose and two times concentrated oligomycin (final concentration of 1μg/μl) or 2DG (final concentration of 10mM) was added. Solution provided by the kit, lysing instantaneously the cells was added at the indicated time after addition of oligomycin/2DG.

Nude mice tumorigenicity and immunohistochemistry

$1x10^6$ cells suspended in 300 μL of serum-free DMEM supplemented with insulin-transferrin-selenium (Gibco) were s.c. injected into the backs of 6wk-old female athymic nude mice (Harlan). The mice were housed under strict pathogen-free conditions and given sterile food and water. Animals were housed 5 per cage. When the tumor reached $70mm^3$, mice were treated twice a day by gavage for ten days with vehicle (methyl cellulose 5g/l, Tween 1g/l) or with MCT1/2 inhibitor (200mg/kg/day, dissolved in the vehicle). The tumor volume was determined by using the formula: 0.52 x L x W x H, where L represents length, W the width, and H the height. Tumors were collected for immunohistochemical analysis. Tumor sections were incubated with antibodies to P-AMPK or P-ACC for 1.5h followed by incubation with antirabbit IgG-HRP antibodies. Analysis was performed with a Leica microscope (20x objective).

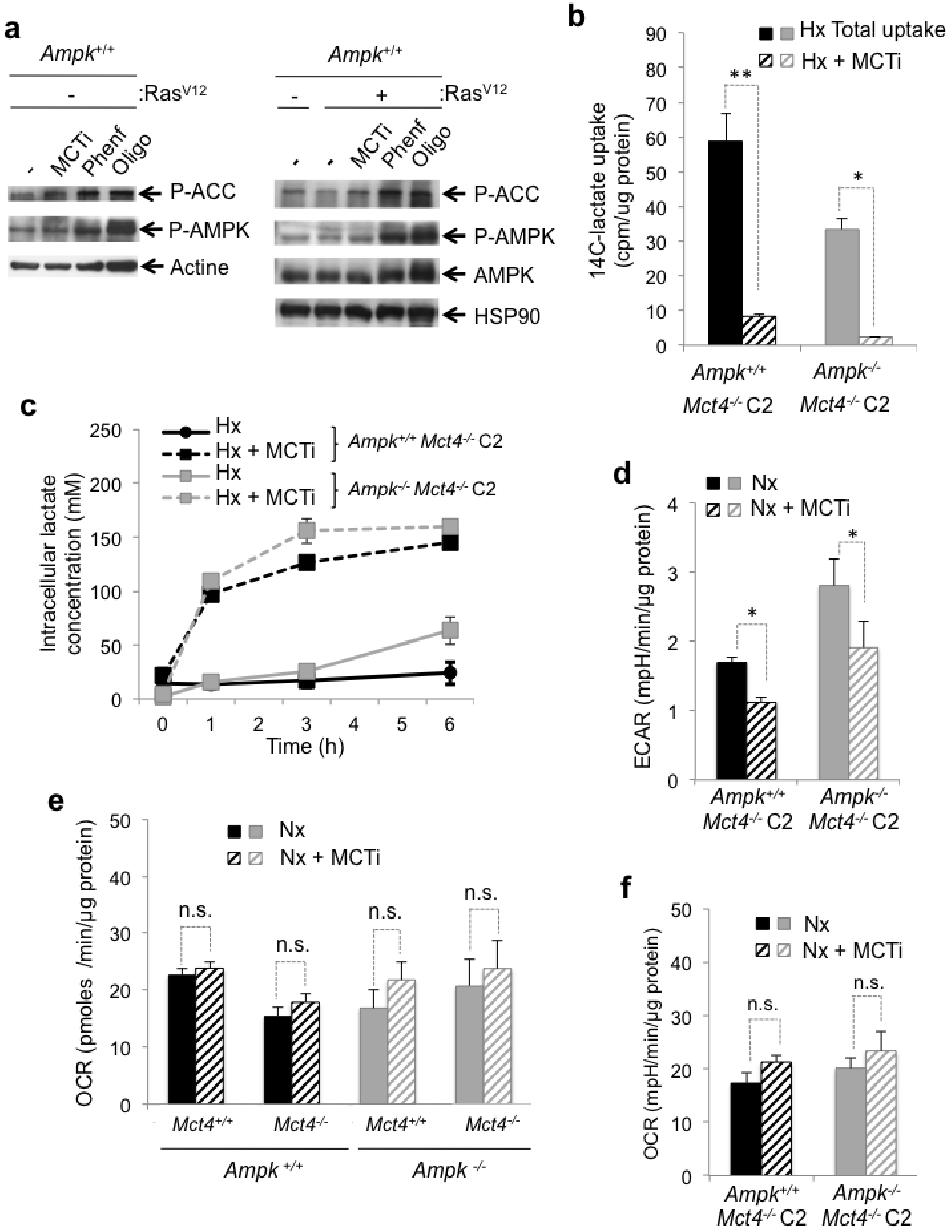

Supplementary Figure S1: The pharmacological inhibitor of MCT1 (MCTi) in *Ampk*$^{+/+}$ and *Ampk*$^{-/-}$ MEFs expressing (*Mct4*$^{+/+}$) or not (*Mct4*$^{-/-}$) MCT4 (clone#2 (C2)) showed reduced lactate transport. (**a**) Cell lysates of *Ampk*$^{+/+}$ MEFs transformed (+) or not (-) by Ras^{V12} were analyzed by immunoblotting for total AMPK (AMPK), for phosphorylation of AMPK (Thr172) and of ACC (Ser79) after 24h of treatment with 300nM MCTi, 50µM phenformin, or 1µM oligomycin. Actine or

HSP90 were used as loading controls. (**b**) Lactate uptake in the *Ampk*$^{+/+}$ (*Ampk*$^{+/+}$, black bar) and *Ampk*$^{-/-}$ (*Ampk*$^{-/-}$, grey bar) MEF (C2) expressing (*Mct4*$^{+/+}$) or not (*Mct4*$^{-/-}$) MCT4, in the absence (total uptake, filled bars) or presence of 300nM MCTi (+ MCTi, hatched bars) in hypoxia 1% O_2 (Hx) for 48h. Uptake was conducted at 3-min time-points in duplicate. The mean±S.E.M. is representative of two independent experiments carried out in triplicate. (**b**) The time-course of the intracellular lactate concentration in response to addition of glucose (25mM) in *Ampk*$^{+/+}$ (*Ampk*$^{+/+}$) (C2) expressing (*Mct4*$^{+/+}$) or not (*Mct4*$^{-/-}$) MCT4 in the presence of DMSO (solid line) or MCTi (300nM, dotted line) in hypoxia 1% O_2 (Hx). The mean±S.E.M. is representative of three independent experiments carried out in triplicate. (**c**) The extracellular acidification rate (ECAR) of *Ampk*$^{+/+}$ (*Ampk*$^{+/+}$, black bar) and *Ampk*$^{-/-}$ (*Ampk*$^{-/-}$, grey bar) MEF (C2) not expressing MCT4 (*Mct4*$^{-/-}$) with DMSO (filled bars) or 300nM MCTi (+ MCTi, hatched bars) in normoxia (Nx) was evaluated after injection of glucose (10mM) with a Seahorse XF. The mean±S.E.M. is representative of three independent experiments carried out in quadruplicate. (**d**) The oxygen consumption rate (OCR) was measured, after glucose injection (10mM), in real time with a Seahorse XF for *Ampk*$^{+/+}$ (*Ampk*$^{+/+}$, black bars) and *Ampk*$^{-/-}$ MEF (*Ampk*$^{-/-}$, grey bars) expressing (*Mct4*$^{+/+}$) or not (*Mct4*$^{-/-}$) MCT4 in normoxia (Nx) in the absence (filled bars) or presence (+ MCTi, 300nM, hatched bars) of the MCT inhibitor. Non significant (n.s.). The mean±S.E.M. is representative of three independent experiments carried out in quadruplicate. (**e**) The oxygen consumption rate (OCR) was measured, after glucose injection (10mM), in real time with a Seahorse XF for *Ampk*$^{+/+}$ (*Ampk*$^{+/+}$, black bar) and *Ampk*$^{-/-}$ (*Ampk*$^{-/-}$, grey bar) MEFs (C2) not expressing MCT4 (*Mct4*$^{-/-}$) with DMSO (filled bar) or 300nM MCTi (+ MCTi, hatched bar) in normoxia (Nx). Non significative (n.s.). The mean±S.E.M. is representative of three independent experiments carried out in quadruplicate.

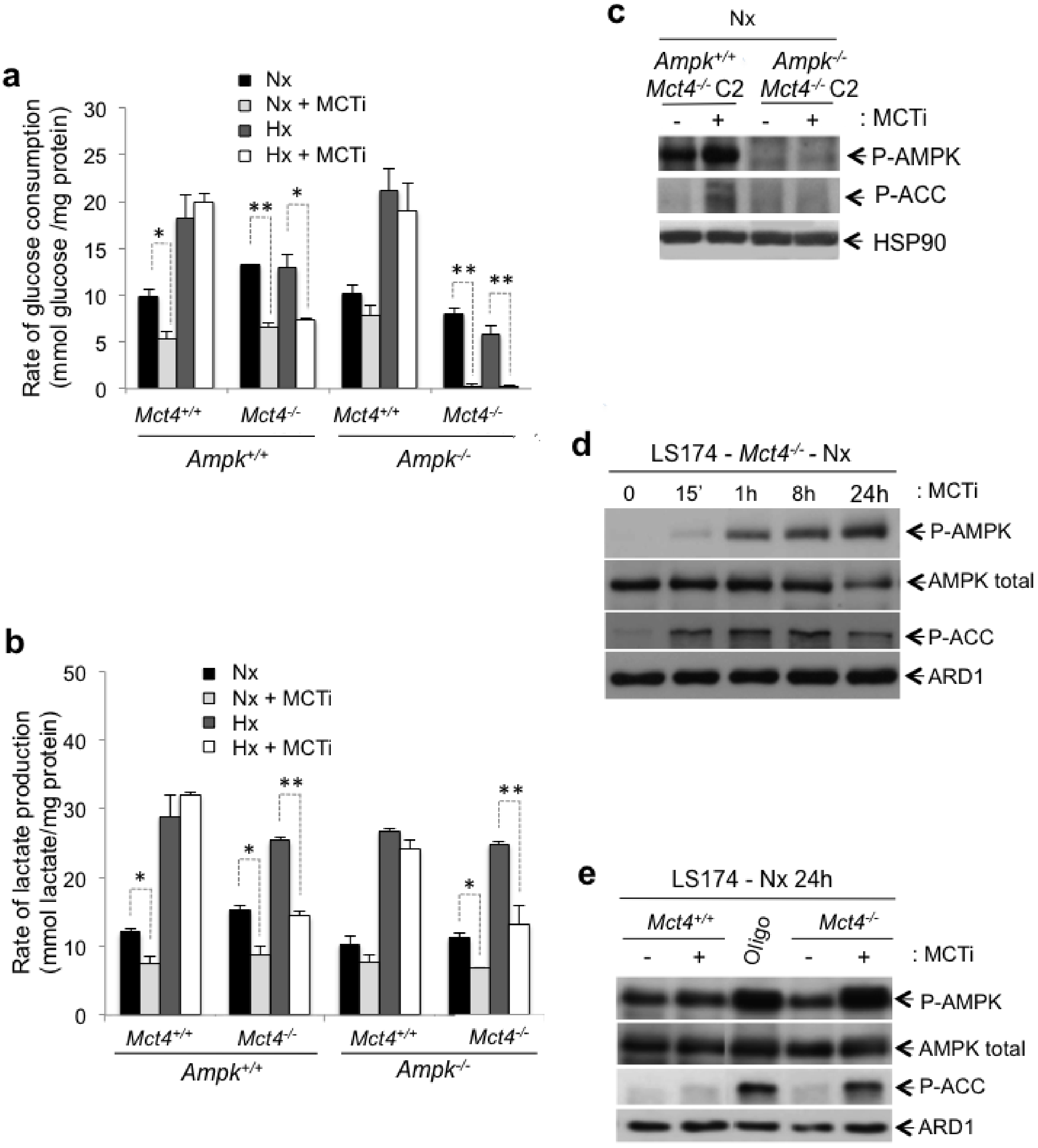

Supplementary Figure S2: Pharmacological inhibition of MCT1 (MCTi) in *Ampk*$^{+/+}$ and *Ampk*$^{-/-}$ MEFs expressing (*Mct4*$^{+/+}$) or not (*Mct4*$^{-/-}$) MCT4 showed reduced lactate transport and glycolysis. (**a**) *Ampk*$^{+/+}$ (*Ampk*$^{+/+}$) and *Ampk*$^{-/-}$ (*Ampk*$^{-/-}$) MEFs expressing (*Mct4*$^{+/+}$) or not (*Mct4*$^{-/-}$) MCT4 cultured in normoxia (Nx) or hypoxia 1% O_2 (Hx) in the absence or presence (+ MCTi, 300nM) of the MCT inhibitor for 48h. Cells were rinsed and 25mM glucose was added (T_{0h}). The rate of glucose consumption was measured in the supernatant (100µl) between T_{1h} and T_{6h} using the YSI 2300 Analyzer. The mean±S.E.M. is representative of two independent experiments carried out in triplicate. (**b**) *Ampk*$^{+/+}$ (*Ampk*$^{+/+}$) and *Ampk*$^{-/-}$ (*Ampk*$^{-/-}$) MEFs expressing (*Mct4*$^{+/+}$) or not (*Mct4*$^{-/-}$) MCT4 cultured

in normoxia (Nx) ou hypoxia 1% O_2 (Hx) in the absence or presence (+ MCTi, 300nM) of the MCT inhibitor for 48h. Cells were rinsed and 25mM glucose was added (T_{0h}). The rate of lactate production was measured in the supernatant (100µl) between T_{1h} and T_{6h} using the YSI 2300 Analyzer. The mean±S.E.M. is representative of two independent experiments carried out in triplicate. (**c**) Cell lysates of *Ampk*$^{+/+}$ (*Ampk*$^{+/+}$) and *Ampk*$^{-/-}$ (*Ampk*$^{-/-}$) MEF (C2) expressing (*Mct4*$^{+/+}$) or not (*Mct4*$^{-/-}$) MCT4 in normoxia (Nx), in the absence (-) or presence (+) of 300nM of MCTi, were analyzed by immunoblotting for Phospho-AMPK (P-AMPK) and Phospho-ACC (P-ACC). Detection of HSP90 was used as a loading control. (**d**) Cell lysates of *Mct4*-knockout (*Mct4*$^{-/-}$) LS174 cells in the presence of MCTi (300nM) for 15min, 1h, 8h and 24h in normoxia (Nx) were analyzed by immunoblotting for Phospho-AMPK (P-AMPK), AMPK total and Phospho-ACC (P-ACC). Detection of ARD1[69] was used as a loading control. (**e**) Cell lysates of LS174 cells expressing (*Mct4*$^{+/+}$) or not (*Mct4*$^{-/-}$) MCT4 in the absence (-) or presence (+) of MCTi (300nM), in the presence of oligomycine (Oligo - 1µM) in normoxia (Nx) for 24h were analyzed by immunoblotting for Phospho-AMPK (P-AMPK), AMPK total and Phospho-ACC (P-ACC). Detection of ARD1 was used as a loading control.

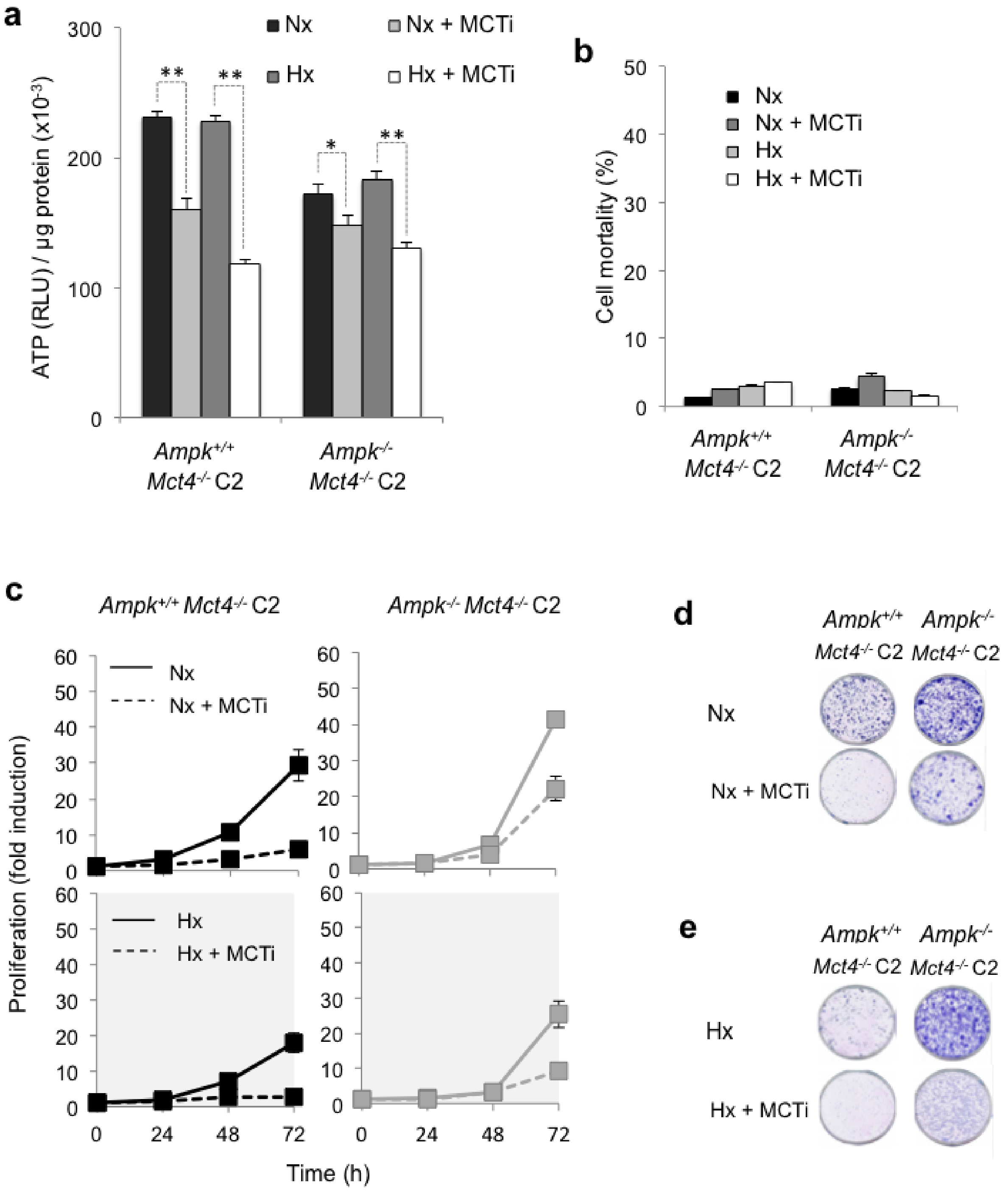

Supplementary Figure S3: Pharmacological inhibition of MCT1 (MCTi) in combination with knockdown of *Mct4* (*Ampk*$^{+/+}$*Mct4*$^{-/-}$ and *Ampk*$^{-/-}$*Mct4*$^{-/-}$ MEFs) (C2) decreased ATP levels and proliferation independently of the presence or absence of AMPK but did not alter cell viability. (**a**) The total cellular ATP level in *Ampk*$^{+/+}$ (*Ampk*$^{+/+}$) and *Ampk*$^{-/-}$ (*Ampk*$^{-/-}$) MEFs (C2) expressing (*Mct4*$^{+/+}$) or not (*Mct4*$^{-/-}$) MCT4, incubated in normoxia (Nx) and in hypoxia 1% O_2 (Hx) for 48h in the absence or presence (+ MCTi, 300nM) of MCTi and standardized to the protein content for each

condition. The mean±S.E.M. is representative of three independent experiments carried out in quadruplicate. (**b**) Cell mortality of *Ampk*$^{+/+}$ (*Ampk*$^{+/+}$) and *Ampk*$^{-/-}$ (*Ampk*$^{-/-}$) MEFs (C2) expressing (*Mct4*$^{+/+}$) or not (*Mct4*$^{-/-}$) MCT4, incubated in normoxia (Nx) or hypoxia 1% O_2 (Hx) for 48h in the absence or presence (+ MCTi, 300nM) of the MCT inhibitor. Cell mortality (%) was evaluated by Trypan blue exclusion. The mean±S.E.M. is representative of three independent experiments carried out in duplicate. (**c**) *In vitro* exponential growth of *Ampk*$^{+/+}$ (*Ampk*$^{+/+}$) and *Ampk*$^{-/-}$ (*Ampk*$^{-/-}$) MEFs (C2) not expressing (*Mct4*$^{-/-}$) MCT4 incubated for up to 72h in normoxia (Nx – top panel) or hypoxia 1% O_2 (Hx – bottom panel) in the absence or presence (+ MCTi, 300nM) of the MCT inhibitor. The mean±S.E.M. is representative of three independent experiments carried out in duplicate. (**d**) Clonal growth in normoxia (Nx) of *Ampk*$^{+/+}$ (*Ampk*$^{+/+}$) and *Ampk*$^{-/-}$ (*Ampk*$^{-/-}$) MEFs (C2) cells not expressing (*Mct4*$^{-/-}$) MCT4, in the absence or presence (+ MCTi, 300nM) of the MCT inhibitor for 8 days before staining for visualization of the colonies. (**e**) Clonal growth in hypoxia 1% O_2 (Hx) of *Ampk*$^{+/+}$ (*Ampk*$^{+/+}$) and *Ampk*$^{-/-}$ (*Ampk*$^{-/-}$) MEFs (C2) not expressing (*Mct4*$^{-/-}$) MCT4, in the absence or presence (+ MCTi, 300nM) of the MCT inhibitor for 8 days before staining for visualization of the colonies.

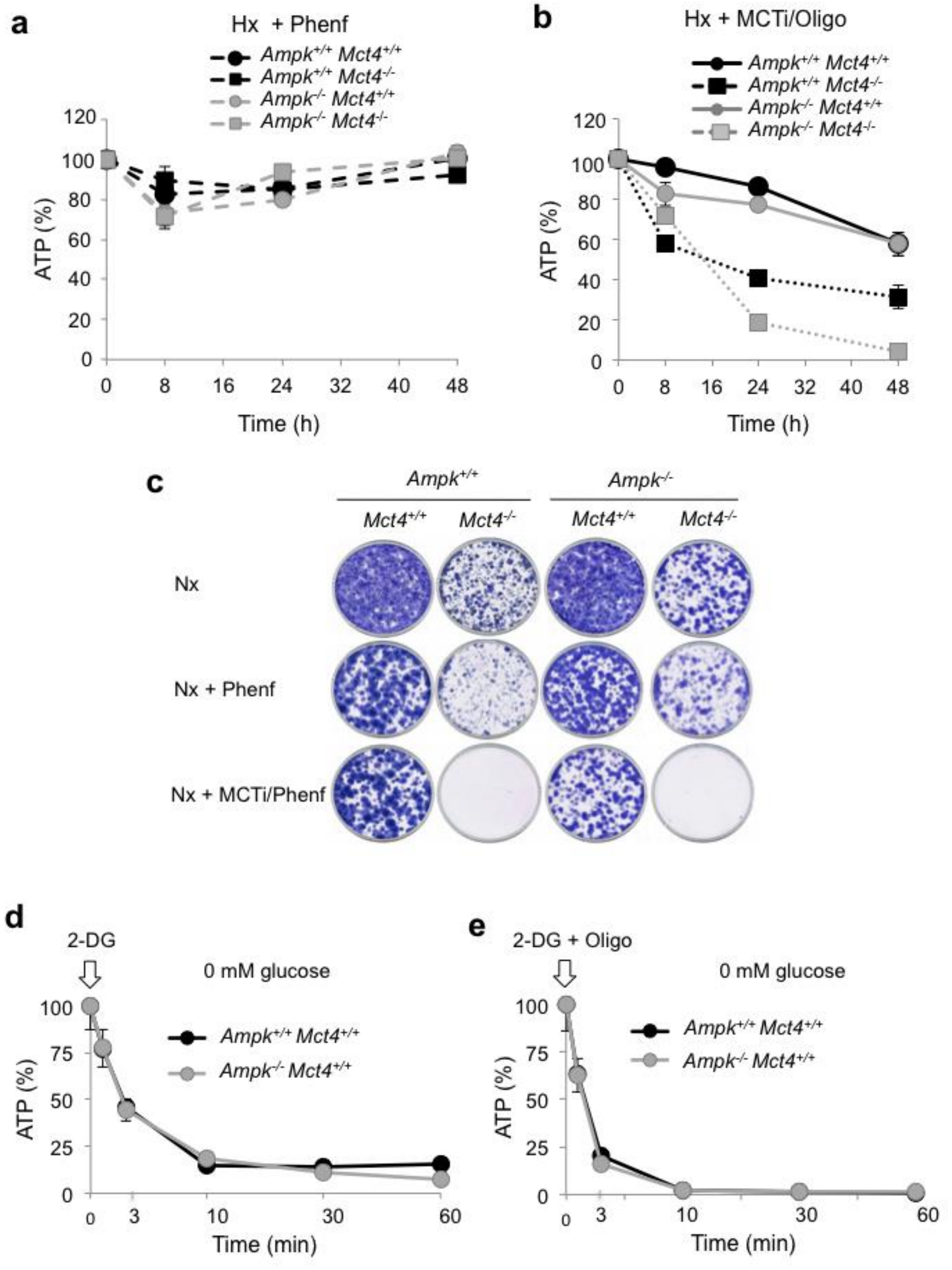

Supplementary Figure S4: AMPK is not sufficient to guaranty viability in response to MCT1 inhibition (MCTi) and *Mct4* knockout (*Mct4*$^{-/-}$) combined with phenformin (Phenf). (**a**) The total cellular ATP level in *Ampk*$^{+/+}$ (*Ampk*$^{+/+}$) and *Ampk*$^{-/-}$ (*Ampk*$^{-/-}$) MEFs expressing (*Mct4*$^{+/+}$) or not (*Mct4*$^{-/-}$) MCT4, incubated in hypoxia 1% O_2 (Hx) up to 48h in the presence of phenformine (+ Phenf, 50 μM), standardized to the protein content for each condition and normalized to initial cellular ATP

level (%). The mean±S.E.M. is representative of three independent experiments carried out in quadruplicate. (**b**) The total cellular ATP level in *Ampk*$^{+/+}$ (*Ampk*$^{+/+}$) and *Ampk*$^{-/-}$ (*Ampk*$^{-/-}$) MEFs expressing (*Mct4*$^{+/+}$) or not (*Mct4*$^{-/-}$) MCT4, incubated in hypoxia 1% O_2 (Hx) for up to 48 h in the presence of a combination of MCTi and oligomycin (MCTi/Oligo), 300nM and 1µM, respectively, standardized to the protein content for each condition and normalized to initial cellular ATP level (%). The mean±S.E.M. is representative of two independent experiments carried out in quadruplicate. (**c**) Clonal growth in normoxia (Nx) of *Ampk*$^{+/+}$ (*Ampk*$^{+/+}$) and *Ampk*$^{-/-}$ (*Ampk*$^{-/-}$) MEFs expressing (*Mct4*$^{+/+}$) or not (*Mct4*$^{-/-}$) MCT4, in the absence or presence (+ Phenf, 50µM) of phenformine in combination with MCTi (+ MCTi/Phenf, 300nM) for 8 days before staining for visualization of the colonies. (**d**) The total cellular ATP level in *Ampk*$^{+/+}$ (*Ampk*$^{+/+}$) and *Ampk*$^{-/-}$ (*Ampk*$^{-/-}$) MEFs expressing (*Mct4*$^{+/+}$) MCT4 incubated in normoxia (Nx) in 0mM glucose in the presence of 2-deoxyglucose (2-DG, 10mM) for up to 60min, standardized to the protein content for each condition and normalized to initial cellular ATP level (%). The mean±S.E.M. is representative of two independent experiments carried out in quadruplicate. (**e**) The total cellular ATP level in *Ampk*$^{+/+}$ (*Ampk*$^{+/+}$) and *Ampk*$^{-/-}$ (*Ampk*$^{-/-}$) MEFs expressing MCT4 (*Mct4*$^{+/+}$) incubated in normoxia (Nx) in 0mM glucose in the presence of a combination of 2-deoxyglucose (+ 2-DG, 10mM) and oligomycin (+ Oligo, 1µM) for up to 60min, standardized to the protein content for each condition and normalized to initial cellular ATP level (%). The mean±S.E.M. is representative of two independent experiments carried out in quadruplicate.
